# The pTau217 interactome in human Alzheimer’s disease brain tissue from APOE3 and APOE4 carriers

**DOI:** 10.1002/alz.090560

**Published:** 2025-01-03

**Authors:** Tomas Kavanagh, Jackeline Ponce, Manon Thierry, Beatrix Ueberheide, Thomas Wisniewski, Eleanor Drummond

**Affiliations:** ^1^ The University of Sydney, Sydney, NSW Australia; ^2^ NYU Grossman School of Medicine, New York, NY USA; ^3^ New York University Grossman School of Medicine, New York, NY USA

## Abstract

**Background:**

Hyperphosphorylated tau (pTau) in Alzheimer’s disease (AD) brain tissue is a complex mix of multiple tau species that are variably phosphorylated on up to 55 epitopes. Emerging studies suggest that phosphorylation of specific epitopes may alter the role of tau. The role of specific pTau species can be explored through protein interaction (“interactome”) studies. These provide knowledge about the role of individual pTau species and critical context for tau‐focused biomarker and drug‐discovery studies. The aim of this study was to analyse the interactome of pTau217, which biomarker studies suggest is one of the earliest accumulating tau species in AD.

**Methods:**

pTau217 interactors were identified in fresh‐frozen human brain tissue from 10 cases of advanced AD using affinity purification‐mass spectrometry. Cases were balanced for ApoE ε3/ε3 and ε4/ε4 genotypes (n = 5 each) to explore if ApoE influences pTau protein interactions. Results were compared to our previous pTauS396/S404 interactome dataset generated using the same cases to determine if individual pTau species have different interactomes.

**Results:**

579 proteins were significantly enriched by pTau217 co‐immunoprecipitation in comparison to control IgG (p < 0.05; fold change >1.5). Of these, 23 proteins were identified as *bona fide* pTau217 interactors (SAINT score > 0.65), including known pTau interactors SQSTM1 and ubiquitin. Phosphorylation analysis of tau enriched by pTau217 vs pTau396/S404 co‐immunoprecipitation confirmed enrichment of a different pools of tau, with pTau217 displaying fewer phosphorylated epitopes, hinting that pTau217 may be an earlier generated species. Despite these differences, 15 *bona fide* pTau217 tau interactors also interacted with pTauS396/S404 suggesting close similarities in interactomes (Fisher’s exact p = 2.3 × 10^‐14^). Common interactors notably included five subunits of an E3 ubiquitin ligase not previously been linked to tau or AD. 46 and 28 pTau217 interactors were identified in ApoE ε3/ε3 and ApoE ε4/ε4 cases respectively and these significantly overlapped (16 common interactors; Fisher’s exact p = 4.3 × 10^‐24^).

**Conclusions:**

pTau217 interacts with many similar proteins to pTauS396/S404. Our results highlight a strong interaction between multiple pTau species and a novel E3 ubiquitin ligase, which may have an important role in AD and potentially tau degradation.

